# Evaluation of interventions for informed consent for randomised controlled trials (ELICIT): developing a core outcome set

**DOI:** 10.1186/1745-6215-16-S2-P53

**Published:** 2015-11-16

**Authors:** Katie Gillies, Cynthia Fraser, Vikki Entwistle, Shaun Treweek, Paula Williamson, Marion Campbell

**Affiliations:** 1 Health Services Research Unit, University of Aberdeen, Aberdeen, UK; 2 Department of Biostatistics, University of Liverpool, Liverpool, UK

## 

The process of obtaining informed consent for participation in randomised controlled trials (RCTs) was established as a mechanism to protect participants against undue harm from research and allow people to recognise any potential risks, or benefits, associated with the research. A number of interventions have been put forward to improve this process. Outcomes reported in trials of interventions to improve the informed consent process for decisions about trial participation tend to focus on ‘understanding’ of trial information and recruitment to the parent study. Yet there is a lack of clarity regarding whether these are the ‘right’ outcomes to measure and which outcomes matter (to whom) and why.

The ELICIT project aims to develop a core outcome set for the evaluation of interventions intended to improve how people make decisions about whether to participate in RCTs of healthcare interventions

The ELICIT project will adopt and adapt methodology previously developed and used in projects developing core outcome sets. Specifically, the work will consist of four stages: 1. A systematic methodology review of existing outcome measures of trial informed consent interventions; 2. Interviews with key stakeholders to explore additional outcomes relevant for trial participation decisions; 3. A Delphi study to refine the core outcome set for evaluation of trial informed consent interventions; and 4. A consensus group meeting to finalise the core outcome set.

This presentation will discuss the key issues relevant for this work and present data generated to date from the systematic literature review, which is reviewing both evidence from explanatory, exploratory and philosophical literature.

